# Identification of key genes and pathways in syphilis combined with diabetes: a bioinformatics study

**DOI:** 10.1186/s13568-020-01009-3

**Published:** 2020-04-27

**Authors:** Wei Li, Chunyi Luo, Xiaoping Xie, Yongjian Xiao, Feijun Zhao, Jialun Cai, Xiangping Zhou, Tiebing Zeng, Bo Fu, Yimou Wu, Xinhua Xiao, Shuangquan Liu

**Affiliations:** 1 Department of Clinical Laboratory, The First Affiliated Hospital of University of South China, No. 69, Chuanshan Road, Shigu District, Hengyang City, 421000 Hunan China; 2 Department of Clinical Laboratory, The Second Affiliated Hospital of University of South China, Hengyang, Hunan China; 3 Institute of Pathogenic Biology and Key Laboratory of Special Pathogen Prevention and Control of Hunan Province, University of South China, Hengyang, Hunan China; 4 Department of Endocrinolog, The First Affiliated Hospital of University of South China, No. 69, Chuanshan Road, Shigu District, Hengyang City, Hunan 421000 China

**Keywords:** *Treponema pallidum*, Syphilis, Diabetes mellitus, Bioinformatics

## Abstract

We noticed that syphilis patients seem to be more susceptible to diabetes and the lesions often involve the kidneys, but the pathogenesis is not yet completely understood. In this study, microarray analysis was performed to investigate the dysregulated expressed genes (DEGs) in rabbit model of syphilis combined with diabetes. A total of 1045 genes were identified to be significantly differentially expressed, among which 571 were up-regulated and 474 were down-regulated (≥ 2.0fold, p < 0.05). Using the database visualization and integration discovery for the Kyoto Encyclopedia of Gene and Genome (KEGG) pathway enrichment analysis. The downregulated DEGs were significantly enriched for biosynthesis of antibiotics, carbon metabolism and protein digestion, while the upregulated DEGs were mainly enriched for cancer and PI3K-Akt signaling pathway. Molecular Complex Detection (MCODE) plugins were used to visualize protein–protein interaction (PPI) network of DEGs and Screening for hub genes and gene modules. ALB, FN1, CASP3, MMP9, IL8, CTGF, STAT3, IGF1, VCAM-1 and HGF were filtrated as the hub genes according to the degree of connectivity from the PPI network. To the best of our knowledge, this study is the first to comprehensively identify the expression patterns of dysregulated genes in syphilis combined with diabetes, providing a basis for revealing the underlying pathogenesis of syphilis combined with diabetes and exploring the goals of therapeutic intervention.

## Introduction

Diabetes mellitus (DM) is a chronic metabolic disease characterized by either absolute and/or relative deficiency of insulin production or action or both. Cardiovascular and cerebrovascular diseases, renal failure, retinal and nerve damage are common complications of DM. After cardiovascular disease and cancer, DM has become the third non-communicable disease (Punthakee et al. 2018). Syphilis is a chronic, systemic sexually transmitted disease caused by infection with *Treponema pallidum* spp. *pallidum* (*T. pallidum*) that remains a public health problem, with an estimated 12 million new cases per year worldwide (Zhang et al. 2014; Luo et al. 2017). The clinical manifestations of syphilis are diverse, and advanced syphilis can lead to a wide range of syndromes. The inability to independently culture and genetically manipulates *Treponema pallidum* has hindered our understanding of the molecular mechanisms of the pathogenesis of syphilis.

**Fig. 1:**
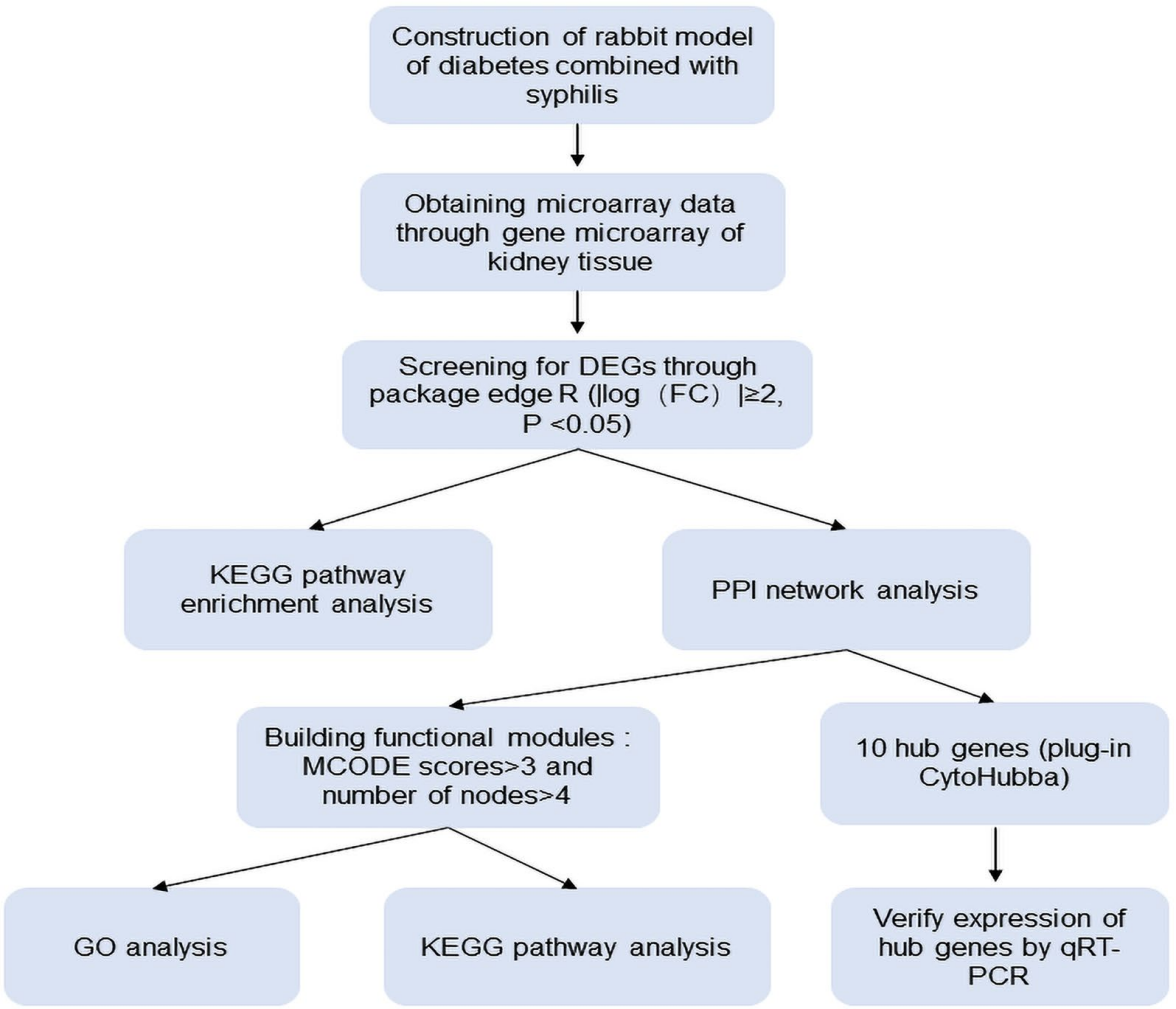
Flowchart for the analysis process

Since syphilis can be cured by antibiotics, the issue of the association between syphilis and DM has rarely been discussed since the 1940s. Although some reports point to the relationship between syphilis and DM, no definitive conclusions have been given (Ortigosa et al. 2016; Comassi et al. 2016; Li et al. 2015). However, recent studies have shown that the prevalence of DM is significantly higher in the individual with neurosyphilis than in the general population. 57.8% of patients with neurosyphilis were diagnosed with DM at the same time, and 80% of patients were diagnosed with DM within 5 years after diagnosis of neurosyphilis (Yang et al. 2014). Another recent retrospective study also reported that 0.8% of DM patients in Spain had syphilis-positive serological results (Esparza-Martin et al. 2013). But the underlying mechanism of this connection remains to be further studied. At present, with the widespread application and development of sequencing, bioinformatics analysis has great advantages in understanding the pathophysiology of syphilis with diabetes on the basis of genetics. In order to understand the pathogenesis of syphilis combined with DM involving the kidney, we performed gene microarray to analyze the expression profiles of genes in the syphilis combined with DM. KEGG and PPI network analysis were performed to predict potential signaling pathways and molecular mechanism of DEGs, provided new evidence to comprehend the potential mechanisms and found therapeutic targets. The workflow of the detailed analysis is illustrated in Fig. 1.

## Materials and methods

### Ethics statement

All animal protocols were approved by the Committee on the Ethics of Animal Experiments of University of South China (Hengyang, China) (permit no.2015-001) and were performed strictly with the Guide for the Care and Use of Laboratory Animals of the National Institutes of Health.

### Animal preparation and grouping

29 New Zealand male rabbits (2.00 ± 0.35 kg) were derived from the Experimental Animal Center of University of South China with negative *T. pallidum* particle agglutination tests (TPPA) and rapid plasma reagin test (RPR) and normal blood glucose. After 1 week of adaptive feeding, the animals were randomly assigned to two groups, normal control group (Group C, n = 3) and syphilis combined with DM group (Group H, n = 3). *T.pallidum* Nichols strain was provided by the Department of Clinical Laboratory, Zhongshan Hospital, Xiamen University.

### Construction of animal models

After fasting for 12–16 h, 100 mg of alloxan per kg body weight was dissolved in 0.9% physiological saline to prepare a 5% concentration solution, which was injected through the rabbit ear vein. Control animals received equal amount of physiological saline instead. When fasting blood glucose of the rabbits were above 16.0 mmol/L for more than 1 week, 0.5 ml of 1∙10^7^*T. pallidum* Nichols/mL bacterial suspension was injected into the testis on both sides of the rabbit for construct *T.pallidum* infection model and observe the changes of testicular morphology. Syphilis combined with DM rabbit model was considered successful constructed successfully when both the reactive RPR and TPPA tests were positive. The explicit method can be seen in the previous report (Liu et al. 2015).

### Gene microarray

Total RNA was obtained from rabbit kidney tissue used TRIzol reagent (Invitrogen, USA). After quantifying and assessing RNA integrity, total RNA from each sample was amplified and transcribed into fluorescent cRNA. The labeled cRNA were hybridized to the microarray (4 × 44 K, Agilent Technologies) and the arrays were scanned with an Agilent Scanner (part number G2505C). The acquired array images were analyzed using Agilent feature extraction software (version 11.0.1.1).

### KEGG pathway enrichment analysis

KEGG (http://www.genome.jp/kegg/) is a database of gene function analyses characterized by linking gene lists obtained from fully sequenced genomes with higher levels of systemic function. In order to analyze the DEGs at the functional level, KEGG pathway analysis were performed with DAVID online tool (https://david.ncifcrf.gov/) (Yang et al. 2019a). *p *< 0.05 was considered statistically significant.

### PPI network construction and modular analysis

To identify the interactive relationships among the target genes, we mapped the target genes to the STRING database (V11.0; http://string-db.org/), and only the interactions with a combined score > 0.4 were selected as significant (Chen et al. 2018). Then, Cytoscape software (V3.7.1; http://cytoscape.org/) was used for constructing PPI network (Shannon et al. 2003). According the degree of importance, we screen some significant modules from the modules of PPI network in Cytoscape for further analysis with the plug-in MCODE (Yang et al. 2019b). The main role of MCODE is to cluster and build functional modules in large gene or protein networks and the criteria were set as follows: MCODE scores > 3 and number of nodes > 4 (Liang et al. 2016). GO analysis (http://www.geneontology.org/) was applied to elucidate genetic regulatory networks of three module according to the biological process. KEGG pathway analysis were performed to explore the significant pathways. The top 10 hub genes were selected according to the MCC method using the plug-in CytoHubba.

### RT-qPCR

Total RNA was obtained from rabbit kidney tissue using TRIzol Reagent (Invitrogen, USA). Reverse transcription and amplification were performed by Transcriptor First Strand cDNA Synthesis Kit and SYBR Green I Master (Roche, Germany) according to the manufacturer’s instructions. And primers were designed using primer design software Primer 5.0 (Table 1). PCR amplification was carried out using real-time PCR amplification instrument under the following conditions: 95 °C, 10 min; 40 PCR cycles (95 °C, 10 s; 60 °C, 60 s; 95 °C, 15 s).

**Table 1:** Primer sequences used for RT-qPCR polymerase chain reaction

Genes	Primer (5′ → 3′)
*ALB*	5′-ACAGAGACTCAAGTGTGCCAGT-3′
	5′-GCAAGGTCCGCCCTGTCATC-3′
*FN1*	5′-CAGTGGCATCGAGACAGACAGTG-3′
	5′-AGTGGCATCAAGGGAAGAGGACTC-3′
*CASP3*	5′-CAGTGGCATCGAGACAGACAGTG-3′
	5′-AGTGGCATCAAGGGAAGAGGACTC-3′
*MMP9*	5′-AAGACGCAGACGGTGGATTC-3′
	5′-ACTCACACGCCAGAAGAAGC-3′
*IL8*	5′-TGGCTGTGGCTCTCTTGG-3′
	5′-ATTTGGGATGGAAAGGTGTG-3′
*CTGF*	5′-CTTCCCGAGGAGGGTCAAAC-3′
	5′-GTGGTCCTTGGGCTCATCAC-3′
*STAT3*	5′-GTATAGCCGCTTCCTGCAAGAGTC-3′
	5′-CATCTGCTGCTTCTCCGTCACC-3′
*IGF1*	5′-GCATCCTGTCCTCCTCGCAT-3′
	5′-GTCTTGGGCATGTCGGTGTG-3′
*VCAM*-*1*	5′-TTCCAGCGAGGGTCTACCAGTTC-3′
	5′-CCTTCACTTGTCACCTTCCCACTC-3′
*HGF*	5′-ATTCCTTGTCAGCGTTGGGATTCC-3′
	5′-GTGGGCATCATCGTCGGGATTC-3′
*β*-*actin*	5′-CGTCTTCCCCTCCATCGTG-3′
	5′-GGATGCCTCTCTTGCTCTGG-3′

### Statistical analysis

All data in this study were expressed as mean ± SEM, and the student’s t test were used to evaluate the statistical differences. All experimental data were statistically analyzed using SPSS statistical software (V11.0). *p* < 0.05 were examined statistically significant.

## Results

### Overall differential gene expression profiles

**Fig. 2:**
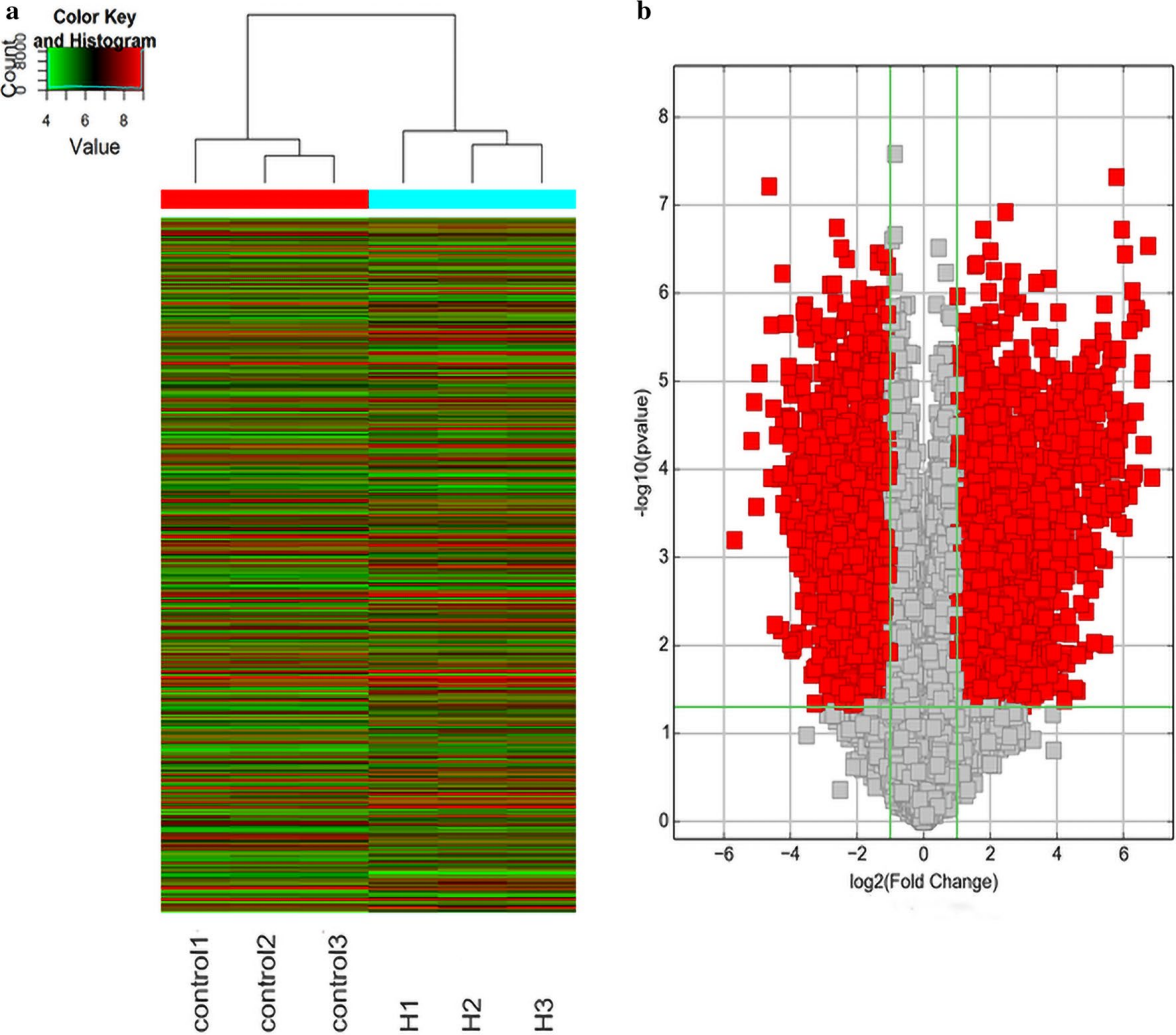
The heat map of the differentially expressed gene and the volcano plot of visualizing differential expression variation between syphilis combined with diabetes and controls. **a** The data are depicted as a data matrix, in which red represents high relative expression and green represents low relative expression. **b** The vertical lines correspond to 2.0-fold up and down, respectively, and the horizontal line represents a p-value of 0.05. Red point in the plot represents the DEGs with statistical significance

The gene expression levels of syphilis combined with DM compared to the normal rabbits was evaluated by microarray analysis. We identified 1045 DEGs altogether (Additional file 1), of which 571 and 474 were upregulated and downregulated respectively in syphilis combined with DM (fold change > 2.0, p < 0.05) (Fig. 2a). The variation of visualizing differential expression between the syphilis combined with DM and normal rabbits were shown with volcano plots (Fig. 2b). The top 30 most significantly differentially expressed genes are shown in Additional file 2: Table S1.

### KEGG pathway analysis

**Fig. 3:**
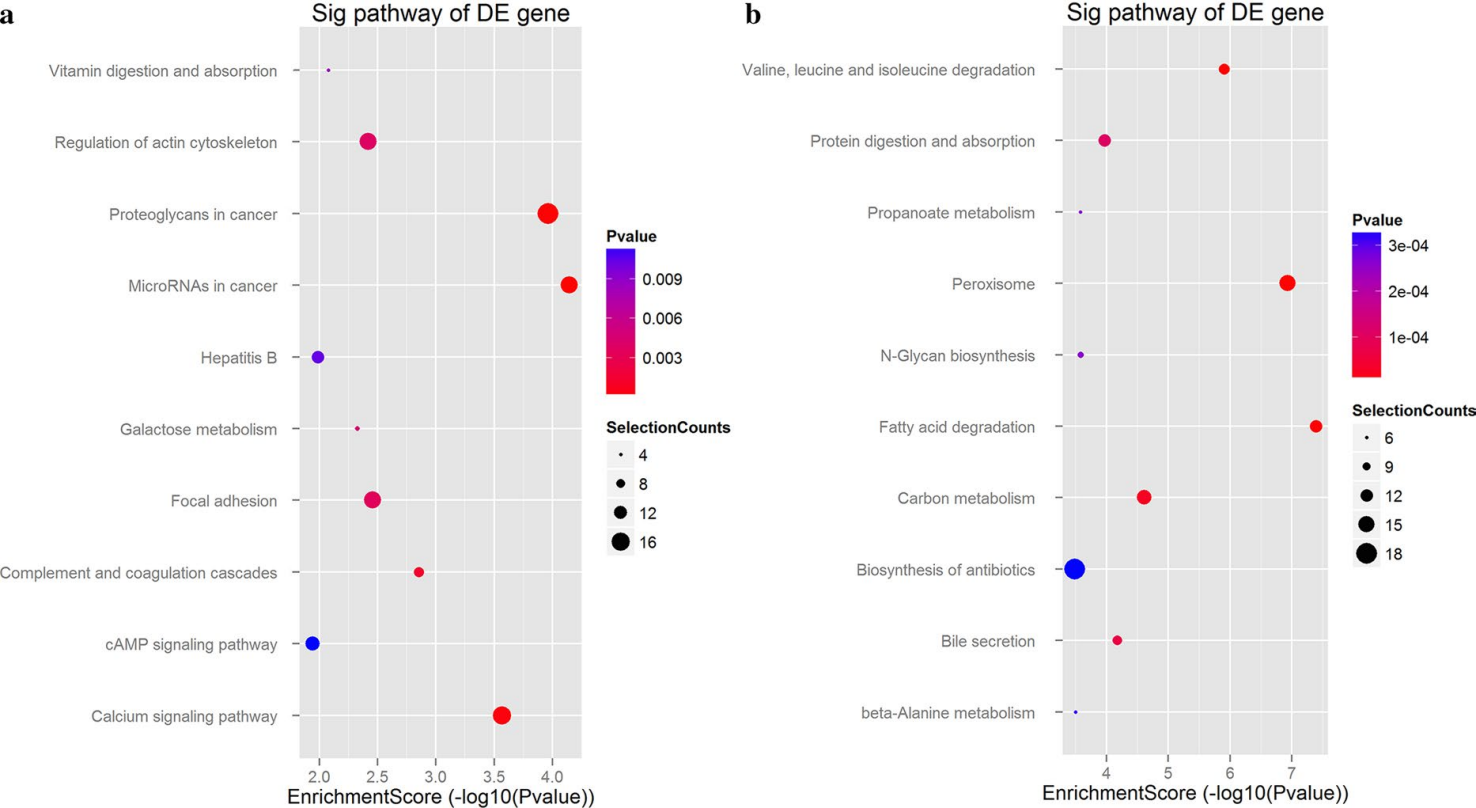
KEGG pathway analysis of the DEGs in syphilis combined with diabetes. **a** For target genes of up-regulated gene. **b** For target genes of down-regulated gene

To further investigate the functional information of DEGs, we performed a KEGG pathway enrichment analysis. The enriched KEGG pathways for the up-regulated target genes involved pathways in cancer, PI3K-Akt signaling pathway, focal adhesion, regulation of actin cytoskeleton, proteoglycans in cancer, cAMP signaling pathway, Epstein-Barr virus infection and calcium signaling pathway (Fig. 3a). For the down-regulated functional genes, the enriched KEGG pathways included biosynthesis of antibiotics, carbon metabolism, protein digestion and absorption, chemical carcinogenesis, biosynthesis of antibiotics, retinol metabolism, peroxisome and arachidonic acid metabolism (Fig. 3b).

### Hub genes and pathways identification with PPI network and modular analysis

**Fig. 4:**
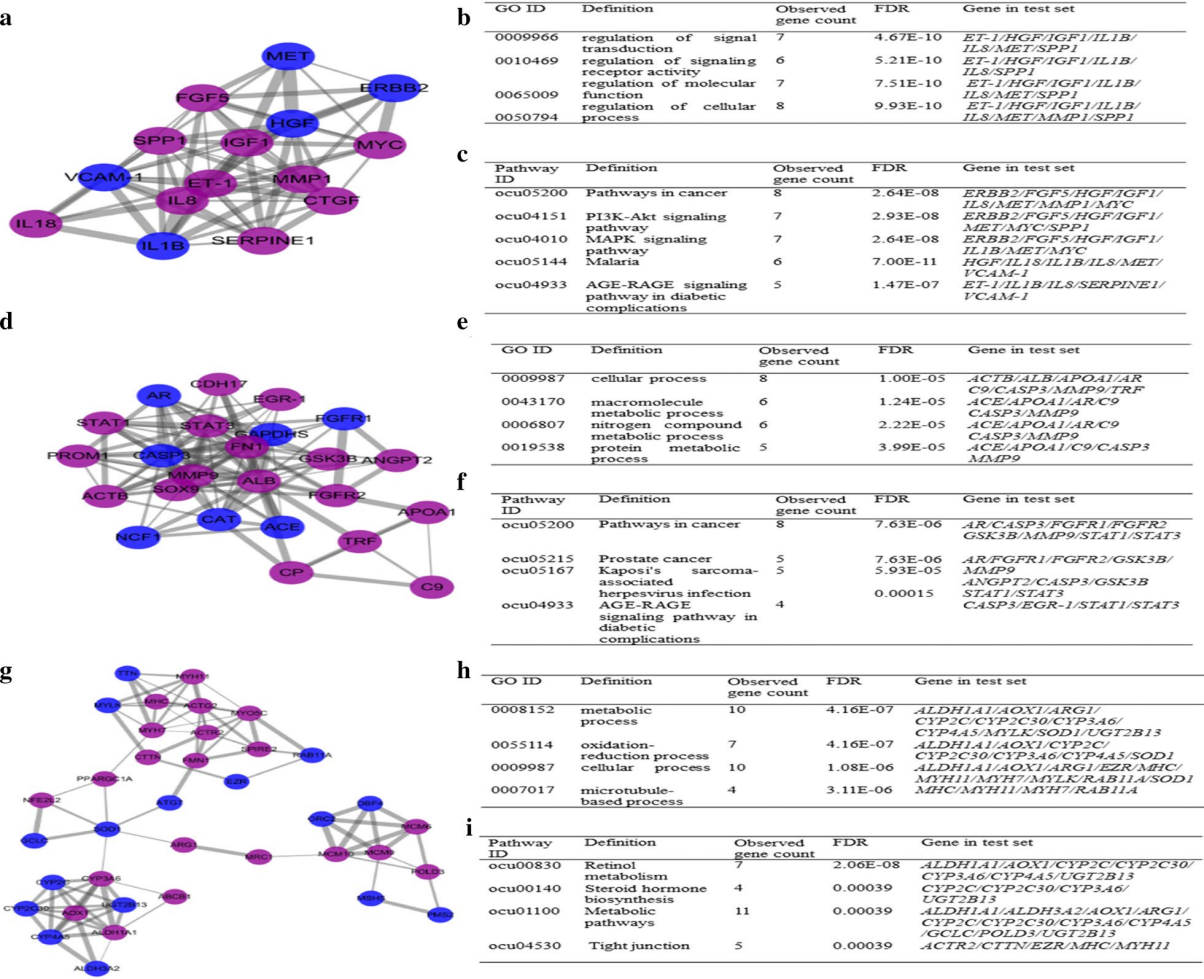
Module analysis of PPI network. **a** module 1; **b** module 2; **g** module 3; Purple represents high relative expression and blue represents low relative expression. GO enrichment analysis of module 1, module 2, and module 3 for **b**, **e** and **h** respectively. The ontology mainly covered the first few important biological processes; KEGG pathway analysis of module 1, module 2, and module 3 for **c**, **f** and **i** respectively

Based on STRING database, plug-ins MCODE was used to carry out module analysis in Cytoscape software. We chose top 3 significant modules from the PPI network complex according to the degree of importance and further analyzed with the plug-in MCODE. PPI network of Module 1 consisted of 71 edges and 15 nodes that was constructed by Cytoscape software (Fig. 4a). GO biological processes analysis results showed that the genes of Module 1 were significantly enriched in regulation of signal transduction, regulation of signaling receptor activity, regulation of molecular function and regulation of cellular process (Fig. 4b). KEGG pathway analysis revealed that genes in module 1 were primarily associated with Pathways in cancer, PI3K-Akt signaling pathway, MAPK signaling pathway, Malaria and AGE-RAGE signaling pathway in diabetic complications (Fig. 4c). GO biological processes analysis showed that Module 2 consisted of 110 edges and 24 nodes (Fig. 4d), which are mainly associated with cellular process, macromolecule metabolic process, nitrogen compound metabolic process and protein metabolic process (Fig. 4e), and that Module 3 consisted of 37 nodes and 90 edges (Fig. 4g), which are mainly associated with metabolic process, oxidation–reduction process, cellular process and microtubule-based process (Fig. 4h). KEGG pathway enrichment analysis indicated that the genes of Module 2 are mainly associated with Pathways in cancer, Prostate cancer, Kaposi’s sarcoma-associated herpesvirus infection and AGE-RAGE signaling pathway in diabetic complications (Fig. 4f), and that the genes of Module 3 are mainly associated with Retinol metabolism, Steroid hormone biosynthesis, Metabolic pathways and Tight junction (Fig. 4i).

### Validation of the top 10 hub genes

**Fig. 5:**
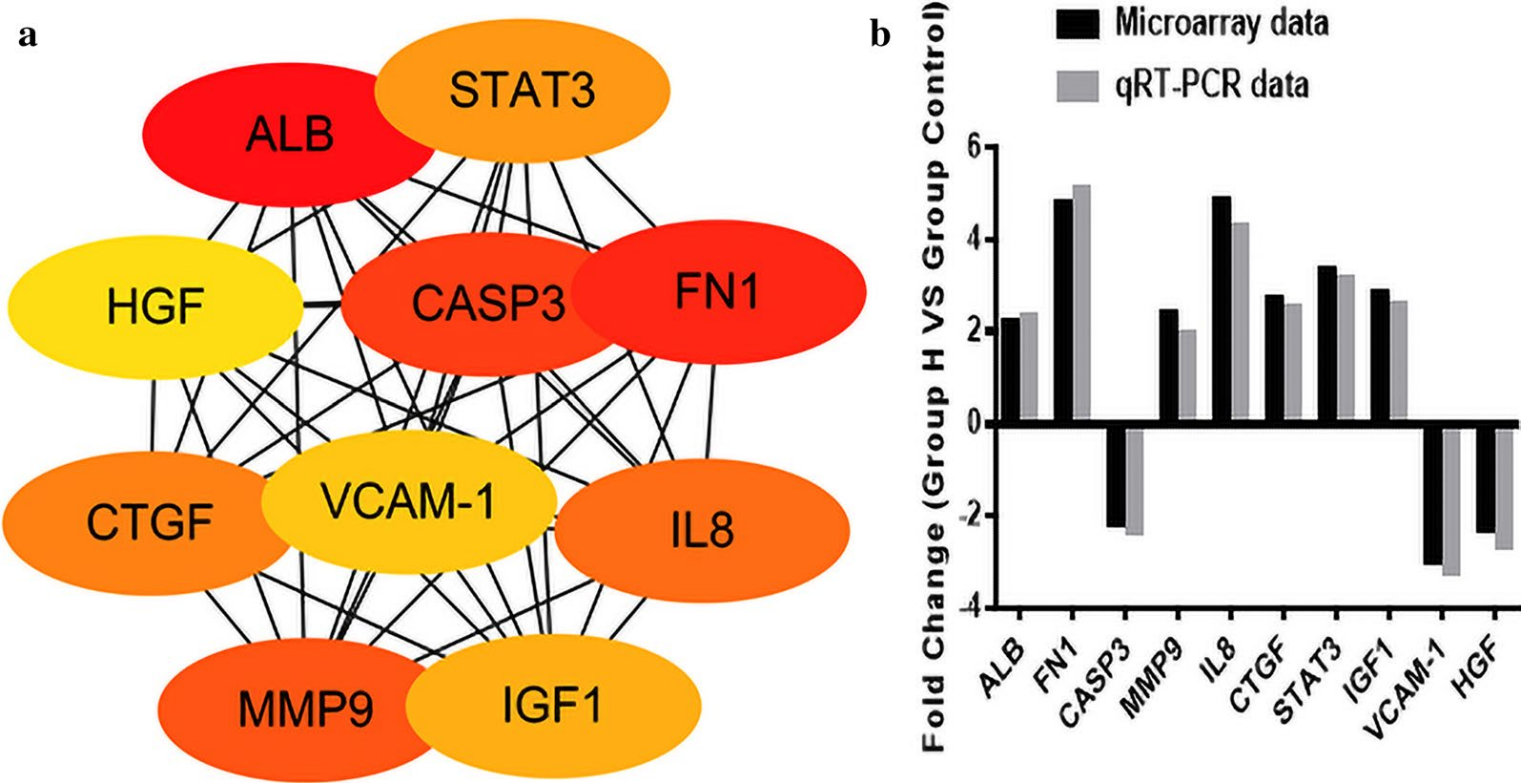
The validation of top 10 hub genes. **a** The more forward ranking is represented by a redder color. **b** The heights of the columns in the chart represent FCs. p < 0.05 was considered to indicate a statistically significant difference

ALB, FN1, CASP3, MMP9, IL8, CTGF, STAT3, IGF1, VCAM-1 and HGF were identified as the top 10 potential hub genes according to the degree score generated by the cytohubba plug-in (Fig. 5a). Gene expression levels were verified using qRT-PCR and compared with Microarray data. The data showed that compared with the control group, the expression levels of CASP3, VCAM-1 and HGF were down-regulated in patients with syphilis and diabetes, while ALB, FN1, MMP9, IL8, CTGF, STAT3 and IGF1 were up-regulated (Fig. 5b). The results are similar to the Microarray data suggesting that the Microarray data is reliable.

## Discussion

The previous reports indicate that comparatively uncomplicated DM or syphilis, there are significant differences in clinical symptoms, treatment plans and treatment effects for Syphilis combined with DM (Comassi et al. 2016; Duarte et al. 2018). Additionally, research have identified that some DM patients can following or meanwhile infect syphilis and often involving kidney. The pathogenesis of syphilis combined with DM remain largely unknown. Analysis of the expression profiles of differential genes may provide new insights into our understanding of the etiology and pathophysiology of syphilis combined with diabetes. Since microarray analysis can simultaneously provide expression profiles of thousands of genes in the human genome, it is widely used to predict potential targets and provides a basis for understanding the molecular mechanisms of DM with syphilis. This study is the first to comprehensively identify the expression patterns of dysregulated genes in syphilis combined with DM and further our understanding differential genes which are associated with the pathogenesis of Syphilis combined with DM.

In this study, we identified 1045 DEGs using gene microarray analysis. Of these, 571 and 474 were found to be significantly up- and down-regulated in the syphilis combined with DM rabbits compared with the controls using bioinformatics analysis. KEGG pathway analysis indicated that several pathways with the highest enrichment scores are microRNAs in cancer, fatty acid degradation, peroxisome, proteoglycans in cancer and calcium signaling pathway. MicroRNAs are key regulators and core players in the regulation of gene expression during cell growth and development at the translational level (Paul et al. 2018). MicroRNAs are involved in the pathogenesis of diabetes and diabetes-related complications (kidney disease, cardiovascular disease, nerve damage, retinopathy) by affecting pancreatic β-cell function and/or insulin resistance (LaPierre and Stoffel 2017; Banerjee et al. 2017; Zhang et al. 2017).

A recent report demonstrated the up-regulation of miR-351 inhibits the PI3K/AKT pathway by reducing FLOT2 in gestational diabetic mice, thereby reducing insulin resistance and hepatic gluconeogenesis (Chen et al. 2019). PI3K activation triggers phosphorylation of PIP2 to PIP3, which phosphorylates AKT through several intermediate kinases to regulate insulin metabolism, resulting in increased glucose uptake and reduced gluconeogenesis. It has been proved that miR-145 (Wen et al. 2014), miR-126 (Ryu et al. 2011) and miR-96 (Jeong et al. 2013) cause impaired insulin signaling and insulin resistance by targeting Insulin Receptor Substrates 1 and miR-152 (Wang et al. 2016), miR-20a-5p (Fang et al. 2016) and miR-19a (Dou et al. 2015) regulates hepatic glycogen synthesis by targeting Phosphatase and Tensin Homolog. Several studies have observed microRNAs play crucial roles in the pathogenesis of syphilis and DM (Sekar et al. 2016; Lu et al. 2017; Singh et al. 2001). It is worth noting that it has recently been found that the sera of patients with syphilis have significantly different expression of multiple microRNAs compared to the serum of non-syphilitic patients (Lu et al. 2017). However, the mechanism of microRNA in the pathogenesis of syphilis needs further study.

We also constructed the PPI network with the DEGs to identify interactions, according the degree of importance, we chose 3 significant modules from the PPI network complex for further analysis using MCODE. A total of 66 nodes and 271 edges were included in three modules, which KEGG pathway enrichment analysis mainly consisted of pathways in cancer, Metabolic pathways,AGE-RAGE signaling pathway in diabetic complications,PI3K-Akt signaling pathway and MAPK signaling pathway. Interestingly, we found that AGE-RAGE signaling pathway appeared twice in the KEGG pathway analysis of the three modules, accounting for 9 hub genes. Advanced glycation end products (AGEs) are complex compounds produced by glycation and oxidation of reducing sugars with proteins or other compounds, primarily associated with inflammatory responses, kidney damage, and DM and its complications (Vlassara and Striker 2013; Bohlender et al. 2005). The receptors for advanced glycosylation end products (RAGE or AGER) are the main receptors of AGEs and are considered to be a pattern recognition receptor. As a signal transduction receptor, RAGE mediates the binding of AGEs and its ligands on the cell surface, triggering the activation of various intracellular signaling pathways involving NF-κB, NADPH oxidase, PI3K-AKT dependent pathway and MAPK, and plays an important role in inflammatory response, vascular calcification, cell proliferation and apoptosis (Frimat et al. 2017; Ramasamy et al. 2015; Davis et al. 2016). The results of these signal transductions were reported to be a possible mechanism for diabetic complications (Khangholi et al. 2016). Inevitably, some limitations should be acknowledged in this study. Firstly, limited to long-term culture of *Treponema pallidum* and lack of effective molecular biology in the syphilis rabbit model, hub genes had no further verifying the potential function and associated pathways to more accurately reflect the pathophysiology of syphilis combined with DM. Secondly, bioinformatical analysis tends to focus on DEGs, which omits some genes with insignificant differential expression but important biological significance, and ignore some valuable information such as the biological characteristics of genes, the relationship between gene regulatory networks and gene functions.

We identified 10 central genes, including FN1, CASP3, MMP9 and IL8. The extracellular matrix (ECM) component of the host is an ideal adhesion target for infection and colonization of many pathogenic bacteria. Fibronectin encoded by FN1 is an important ECM molecule involved in cell proliferation, adhesion and migration. Studies have shown that a variety of *T. pallidum* proteins can bind to fibronectin to mediate *T. pallidum*-host interactions including Tp0136, Tp0155 and Tp0483 (Brinkman et al. 2008; Cameron 2003; Cameron et al. 2004). MMP degrades ECM proteins, leading to tissue remodeling and helping cells migrate. In addition, MMP9 has been found to be involved in the pathogenesis of diabetes and diabetic complications, such as diabetic retinopathy (Mishra et al. 2016). CASP3 participates in the activation cascade of caspases and is responsible for the execution of apoptosis. Studies have found that the outer membrane protein Tp92 of *T. pallidum* causes THP-1 cell apoptosis through the RIPK1/caspase-8/caspase-3 pathway (Luo et al. 2018). In high glucose environment, caspase-3 induced glomerular cell apoptosis is related to mesangial cell damage (Mishra et al. 2005). CASP3 may be a potential target for the treatment of diabetic co-infection with syphilis, but the specific molecular mechanism of CASP3 in diabetic co-infection with syphilis needs to be further studied. The protein encoded by IL8 is a member of the CXC chemokine family and is a major mediator of the inflammatory response. Elevated IL8 levels are present in a variety of diabetic complications, including diabetic retinopathy and diabetes-tuberculosis (Aravindhan et al. 2018; Borilova Linhartova et al. 2018). Evidence suggests that T. pallidum infection induces IL-8 secretion in macrophages (Xu et al. 2019). In addition, the angiogenic response observed during secondary syphilis was induced by the syphilis antigen TpF1 by activating the IL-8 pathway (Pozzobon et al. 2016). Overall, IL8 plays an important role in syphilis and diabetes, but the mechanism of IL8 in diabetic co-infection with syphilis is currently unknown and needs to be studied.

Overall, this study reveals the expression patterns of DEGs in syphilis combined with DM rabbit models by bioinformatical analysis, which provides new insights into the underlying mechanisms of the coexistence of syphilis and diabetes, and thus could lead to new hypotheses about the comorbidities of syphilis combined with DM. The DEGs are involved in several specific hub genes and signaling pathways, which may become targets for therapeutic intervention.

## Supplementary information

**Additional file 1.** Sample information and sequencing data of syphilis combined with diabetes and controls.

**Additional file 2: Table S1.** Top 30 aberrantly expressed genes in microarray analysis.

## Data Availability

Gene microarray data has been submitted to the GEO repository (GSE135053). The entries are scheduled to be released on 27 Jul 2021.The data used to support the findings of this study are available from the corresponding author or supplementary materials upon request. supplementary files:Sample information and sequencing data of syphilis combined with diabetes and controls.
