# Supplementary material for: Identification of key genes and pathways in syphilis combined with diabetes: a bioinformatics study
Source: AMB Express. 2020 Apr 27;10:83. doi: 10.1186/s13568-020-01009-3 (PMC7186291; doi:10.1186/s13568-020-01009-3)
Supplement: Supplementary file 2 — Additional file 2: Table S1. Top 30 aberrantly expressed genes in microarray analysis. [file 13568_2020_1009_MOESM2_ESM.docx]

**Supplementary Table S1. Top 30 aberrantly expressed genes in microarray analysis**

| ProbeName | GeneSymbol | *P-*value | FDR | Fold change | Regulation | Chr |
| --- | --- | --- | --- | --- | --- | --- |
| A_04_P082938 | FGG | 0.000123239 | 0.002692143 | 115.7151619 | up | 15 |
| A_04_P043367 | ASIP | 0.00028106 | 0.003912828 | 51.9697183 | up | 4 |
| A_04_P002582 | SI | 4.19745E-05 | 0.001720302 | 50.151634 | up | 14 |
| A_04_P101952 | KLF5 | 6.97771E-05 | 0.002045876 | 39.5846338 | up | 8 |
| A_04_P004551 | MYH7 | 9.22965E-05 | 0.002356233 | 37.3135521 | up | 17 |
| A_04_P000322 | SLC14A2 | 0.00174211 | 0.010135642 | 35.447725 | up | 9 |
| A_04_P004512 | FGF5 | 2.30723E-05 | 0.001445988 | 34.0197209 | up | 15 |
| A_04_P063152 | MGA | 1.99571E-05 | 0.001392208 | 29.9255094 | up | 17 |
| A_04_P049747 | CHRNB1 | 0.001151648 | 0.007981332 | 29.6248046 | up | 19 |
| A_04_P082867 | CIT | 0.000513045 | 0.005192416 | 27.7232848 | up | 21 |
| A_04_P050673 | CCDC77 | 0.000256371 | 0.003715427 | 27.0858858 | up | 8 |
| A_04_P012756 | CYP2A11 | 0.000113609 | 0.00259189 | 26.2472093 | up | 1 |
| A_04_P012501 | PKD1 | 7.20206E-06 | 0.001068686 | 25.9254052 | up | X |
| A_04_P086994 | NAALAD2 | 6.08517E-08 | 0.000616732 | 24.9694493 | down | 1 |
| A_04_P000146 | SPP1 | 0.032664601 | 0.067797611 | 24.4565105 | up | 15 |
| A_04_P088597 | TIAM1 | 3.76692E-05 | 0.00166231 | 24.0085 | up | 14 |
| A_04_P001656 | DIO1 | 2.29717E-06 | 0.000784781 | 23.7122682 | down | 13 |
| A_04_P082374 | TMEM236 | 0.005826839 | 0.020960671 | 22.2915732 | down | 16 |
| A_04_P095022 | SLC44A5 | 2.14679E-05 | 0.001436037 | 21.7415828 | up | 13 |
| A_04_P094542 | SMEK2 | 1.06598E-05 | 0.001127366 | 20.8454185 | up | 2 |
| A_04_P091207 | PPP1R12B | 1.29515E-05 | 0.001211456 | 20.3158112 | up | 16 |
| A_04_P014367 | MMP1 | 0.016000104 | 0.040891247 | 19.7933898 | up | 1 |
| A_04_P070054 | SEPT6 | 0.000202286 | 0.00334639 | 18.7529319 | up | X |
| A_04_P002066 | CD4 | 0.001850618 | 0.010486013 | 18.7397249 | up | 8 |
| A_04_P083273 | GLDC | 0.000247169 | 18.5863619 | 18.5863619 | down | 1 |
| A_04_P004761 | FGA | 0.000672927 | 0.00596321 | 18.3568562 | up | 15 |
| A_04_P059268 | SLITRK2 | 0.000128554 | 0.002732994 | 17.4756902 | up | X |
| A_04_P072552 | ECT2 | 2.50589E-05 | 0.001500422 | 17.3150158 | down | 14 |
| A_04_P080352 | SEPT11 | 1.71152E-05 | 0.001334328 | 17.2977697 | up | 15 |
| A_04_P062762 | EPHA8 | 0.015463161 | 0.039911494 | 16.8660165 | up | 13 |
